# Psychometric Properties of the Norwegian Version of the Fear of COVID-19 Scale

**DOI:** 10.1007/s11469-020-00454-2

**Published:** 2021-01-20

**Authors:** M. M. Iversen, T. M. Norekvål, K. Oterhals, L. T. Fadnes, S. Mæland, A. H. Pakpour, K. Breivik

**Affiliations:** 1 Centre on Patient-Reported Outcomes, Department of Research and Development, Haukeland University Hospital, Postboks 1400, N-5021 Bergen, Norway; 2 Faculty of Health and Social Sciences, Department of Health and Caring Sciences, Western Norway University of Applied Sciences, Bergen, Norway; 3 Department of Clinical Science, University of Bergen, Bergen, Norway; 4 Department of Global Public Health and Primary Care, University of Bergen, Bergen, Norway; 5 Department of Addiction Medicine, Haukeland University Hospital, Bergen, Norway; 6 Research Unit for General Practice in Bergen, NORCE Norwegian Research Centre, Bergen, Norway; 7 Qazvin University of Medical Sciences, Qazvin, Iran; 8 Department of Nursing, School of Health and Welfare, Jönköping University, Jönköping, Sweden; 9 Regional Centre for Child and Youth Mental Health and Child Welfare, NORCE Norwegian Research Centre, Bergen, Norway

**Keywords:** Psychometrics, Patient-reported outcomes, COVID-19, Fear of COVID-19 Scale, Fear, Norway

## Abstract

To examine the psychometric properties of the Norwegian version of the Fear of COVID-19 Scale (FCV-19S), randomly selected individuals from a larger registry study were invited. We assessed the reliability and validity of the instrument in a sample of 1089 adults in Norway (response rate 73%). Internal consistency measured by Cronbach’s alpha (0.88) was acceptable. Omega alphaHierarchical (*ωt* = 0.69) was lower indicating that the general factor is less reliable, explaining 69% of the total variance. Confirmatory factor analysis indicated that the FCV-19S is not strictly unidimensional. Exploratory graph analysis and confirmatory factor analysis supported a two-factor model (cognitive and somatic fear), which were highly correlated (*r* = 0.84). The Norwegian version of the FCV-19S showed an underlying two-factor structure. However, the high correlation means the two latent factors (cognitive and somatic fear) act as indicators for a second-order general factor and support use of the FCV-19S sum score. The FCV-19S appears to be a valid instrument to assess fear of COVID-19 with good psychometric properties.

**Supplementary Information:**

The online version contains supplementary material available at 10.1007/s11469-020-00454-2.

COVID-19 has extraordinary spreading properties and is causing high rates of both morbidity and mortality (Lipsitch et al. 2020). The coronavirus 2019 (COVID-19) pandemic has, to date, infected more than 10 million individuals and caused more than 500,000 deaths worldwide (nCov2019.live, 1 July 2020). The high infection rate and relatively high mortality rate, as well as limited effective treatment, has led to the COVID-19 pandemic potentially triggering fear and anxiety. The lack of specific robust screening tools to specifically identify an individual’s psychological responses to COVID-19 during the pandemic and the use of traditional assessment tools (e.g. PHQ-9, HADS, GAD-7) may lead to under-diagnosis or over-diagnosis due to poor COVID-19 specific face validity (Ransing et al. 2020; Pakpour et al. 2020a). Therefore, in order to assess psychosocial responses (fear and anxiety) related to COVID-19, the Fear of COVID-19 Scale (FCV-19S) was developed to design appropriate programmes to mitigate fear. The collection of patient-reported information to capture the individual’s fear of COVID-19 is both timely and important (Ahorsu et al. 2020). The current evidence suggests that a psychiatric epidemic is cooccurring with the COVID-19 pandemic (Hossain et al. 2020). In line with this, some evidence suggests that infectious disease-related public health emergencies (epidemics) may increase suicide risk (Zortea et al. 2020). Mental health care and fear of COVID-19 necessitate greater attention during the COVID-19 pandemic.

The prevalence of the COVID-19 pandemic throughout the world means that comparative international research is required. The FCV-19S has been developed as a scale with seven questions and is assumed to have an unidimensional structure. Although the unidimensional structure has been supported by research on several translated versions of the scale (Chang et al. 2020a; Lin et al. 2020; Sakib et al. 2020; Satici et al. 2020), the Eastern European and Israeli version suggests a bi-dimensional structure representing physiological responses (three questions) and emotional responses (four questions), respectively (Bitan et al. 2020; Reznik et al. 2020). Although authors from the New Zealand and Greek studies revealed weaker fit values based on conventional assumptions, they did not modify the models (Tsipropoulou et al. 2020; Winter et al. 2020). The Arabic and Italian versions confirmed an unidimensional structure when the model had been modified to allow for a number of correlations between pairs of residuals (Alyami et al. 2020; Soraci et al. 2020). Since such correlated residuals might signal multidimensionality (Brown 2006), in-depth investigation of the FCV-19S facture structure is warranted.

The FCV-19S offers great potential for international comparative research on the psychosocial responses to COVID-19 as the instrument is translated into many languages and cultures and used in different populations as vulnerable populations, including elderly, children, adolescents and people with pre-existing physical and mental disease (Ahorsu et al. 2020; Bitan et al. 2020; Chang et al. 2020a; Chang et al. 2020b; Chen et al. 2020; Harper et al. 2020; Pakpour et al. 2020a; Reznik et al. 2020; Sakib et al. 2020; Satici et al. 2020; Soraci et al. 2020). Thus, having a Norwegian translation of the FCV-19S is an advantage.

Our study aimed to examine the psychometric properties of the Norwegian version of the FCV-19S and determine the level of fear of COVID-19 in a Norwegian population. As part of construct validity, we tested whether confirmatory factor analysis would detect a one-factor structure in these data. If the fit of the unidimensional model is unsatisfactory, we will follow up with exploratory analysis to analyse the dimensionality of the scale. To test concurrent validity, we hypothesise that the FCV-19S scores are negatively associated with satisfaction with life, and positively associated with the shortened Hopkins symptom checklist (SCL-10), and that it discriminates between men and women and those with high and low socioeconomic status.

## Methods

### Participants

**Fig. 1:**
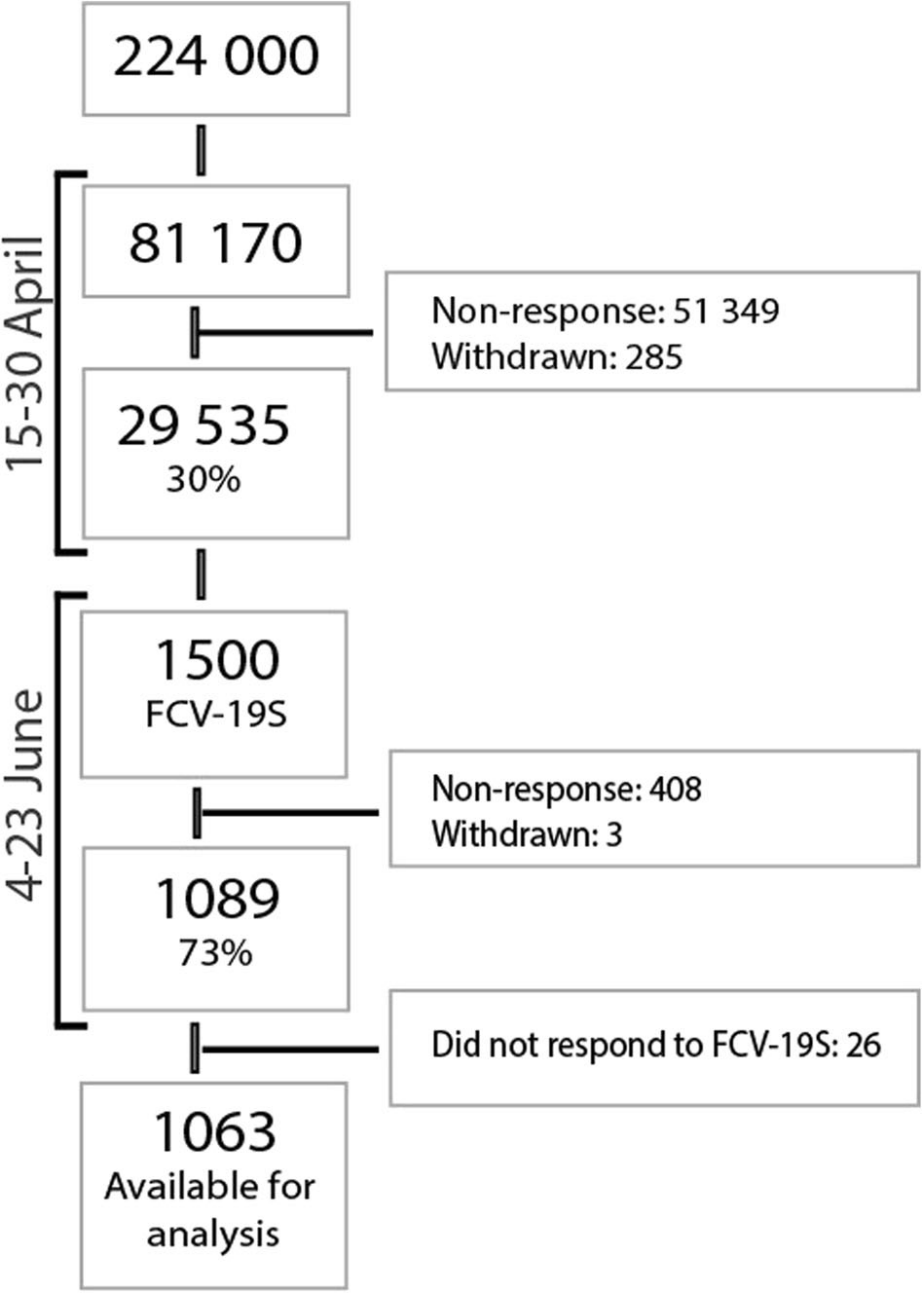
Flowchart of selection and inclusion in the validation study

A representative sample of 81,170 individuals from among 224,000 adult inhabitants in the City of Bergen in Western Norway were invited in April 2020 to participate in a study surveying the effect of the lockdown during the COVID-19 pandemic. The individuals invited to participate were drawn from the Contact and Reservation Registry through the Norwegian Digitalisation Agency. In total, 29,535 individuals consented to participate in the first wave of the study. For the present validation study, 1500 individuals were randomly selected to participate in a follow-up survey. With an anticipated response rate of 30–40%, we estimated that this would give us a satisfactory number of participants for the psychometric analysis. A total of 1089 (73%) consented to participate in this second emailing. As 15 responded to no items in this follow-up survey, and an additional 11 did not respond to the FSV-19S, 1063 individuals were available for analysis (Fig. 1).

### Measurements

#### Characteristics of the Study Population

Demographic information collected included age (in groups), gender, marital status, smoking status, education level (lower secondary school, upper secondary school, college/university less than 4 years, college/university 4 years or more), household income, employment status before the COVID-19 outbreak (working, retired, sick leave, disability pension, seeking employment, student/military service), change of employment status after the COVID-19 outbreak (working from home, laid off, unemployed, new employment, receive wages but not working), sector of employment (healthcare, retail, transport, industry, education, fire-service/police) and Norwegian citizenship. Demographic information was collected during the first and second emailing (Fig. 1).

#### COVID-19 Specific Questions

Questions specifically related to the COVID-19 pandemic over the last 4 weeks included quarantine, social distancing, working from home and/or home schooling, ill with suspected, possible or confirmed COVID-19 and living in the same household as someone with suspected, possible or confirmed COVID-19. This information was collected during the first emailing (Fig. 1).

#### General Health and Meaningfulness

Two global items on general health and meaningfulness were included: ‘How would you rate your general health?’ (1 not at all to 10 to a very high degree), and ‘To what degree do your experience what you do as meaningful?’ (very poor/poor/neither good nor poor/good/very good). This information was collected during the first and second emailing (Fig. 1).

#### The 10-Item Hopkins Symptom Checklist

SCL-10 is a shortened version of the original 25-item symptom checklist (SCL-25) (Hesbacher et al. 1980), and is designed to measure anxiety and depression in large health surveys. The participants are asked to rate, on a scale from 1 to 4, how bothered or distressed they were over the past 14 days by each of the 10 symptoms, four of which address anxiety and six depression. The questionnaire has demonstrated good psychometric properties in previous Norwegian studies (Strand et al. 2003). The SCL-10 was distributed in the second emailing (Fig. 1).

#### Fear of COVID-19 Scale

The FCV-19S is a seven-item scale that assesses the fear of COVID-19. The seven items (e.g. “I am most afraid of corona”) are rated on a 5-point scale from 1 (strongly disagree) to 5 (strongly agree) with scores ranging from 7 to 35. The higher the score, the greater the fear of COVID-19 (Ahorsu et al. 2020). The FCV-19S was distributed in the second emailing (Fig. 1).

### Translation and Cross-Cultural Adaptation of the FCV-19S

A cross-cultural adaptation process was conducted to assure equivalence between the original source and the Norwegian target version of the FCV-19S. The translation process was conducted systematically in six steps including forward and back-translation as recommended (Beaton et al. 2000). The research team encountered some difficulties in translating some phrases in the original English version of the FCV-19S into Norwegian. The issues were discussed with the developer of the FCV-19S for clarity. The persons who participated in the cognitive interviews (*n* = 18) reported that they clearly understood the items and response options. Questions or suggested changes were considered by the research team but did not result in any additional changes to the final version of the instrument. A summary of the overall translation procedure is described in Textbox 1.

**Textbox 1** Steps for translation and cross-cultural adaptation of the Fear of Covid-19 Scale (FSV-19S) into Norwegian

**Table:** 

**Step 1: Forward translation** i. Two forward translations of the English FSV-19S were made by two bilingual translators for whom the target language (Norwegian) was their mother tongue. ii. The translators worked independently and wrote a report (TL1 and TL2) that identified challenging phrases and described their rationale for final translation choices. iii. The two translations were compared by the research team and discrepancies were identified. **• Step 2: Synthesis** i. The research team synthesized the reports (TL1 and TL2) into one consensus version (TL3) and described how they resolved discrepancies. **• Step 3: Back translation** i. Two individuals who had a good understanding of English and also spoke Norwegian fluently independently translated TL3 back into English (TL4 and TL5). Neither of the translators who spoke English as their native language was aware of the original version of the FSV-19S. **• Step 4: Synthesis** i. The research team agreed on the modified Norwegian version of the FSV-19S (TL6). ii. The research team discussed the timing of administration and meaning of certain words and sentences. iii. The instrument developer was contacted in order to clarify issues. **• Step 5: Instrument pilot testing** i. Cognitive interviews were conducted to determine the feasibility and whether the items were understandable. The individuals were asked to comment on the questionnaire items and the amount of time needed to respond. **• Step 6: Revised instrument** i. The researchers evaluated the adapted FSV-19S questionnaire (TL6) and all necessary changes were made.	

### Data Collection

For the first wave of the data collection, an electronic battery of questionnaires was distributed via email through the SurveyXact system on 15 and 16 April 2020. Two reminders were sent, first via text message and then via email. The restrictions imposed at the time included social distancing, closure of schools and universities, as well as sports arenas and other public places where people could potentially gather in large numbers. Employees were encouraged to work from home, and travel restrictions and quarantine and isolation requirements were imposed. There were no changes to restrictions during this first wave of data collection. The survey closed on 30 April 2020. For this validation study, a follow-up emailing was performed on 4 June 2020. A battery of questionnaires including FCV-19S were distributed via email through the SurveyXact system to 1500 randomly selected individuals (Fig. 1). Two reminders were sent, and the survey closed on 23 June 2020. By this time, the restrictions had brought the pandemic under control in Norway, and the restrictions had been eased at the time of the second emailing. Schools had re-opened, and organised sports activities were slowly picking up. However, social distancing and the advice to avoid public transport were still maintained.

### Ethical Considerations

The study was approved by the Norwegian Regional Committee for Ethics in Medical Research (REK 2020/131560). All participants provided informed consent by responding to the emailed survey, and confidentiality and the right to withdraw from the study were assured. The study complied with the ethical principles outlined in the Declaration of Helsinki.

### Data Analyses

The descriptive statistics and correlation analyses were analysed in the IBM SPSS Statistics Version 25.0 software. The remaining analyses were conducted in the R statistical environment (R Development Core Team 2020) using various packages (R Foundation for Statistical Computing n.d.). Reliability was assessed using Cronbach’s alpha, Omegatotal alpha and Omegahierarchical (using two specific factors) which are available in the psych package (Revelle 2014).

Exploratory dimensionality assessment was performed using Exploratory graph analysis available in the EGAnet package (Golino et al. 2020). Exploratory graph analysis is a relatively new and promising statistical method for estimating the number of factors and has demonstrated a good ability to determine the correct number of underlying factors (Golino et al. 2020; Golino and Christensen, 2019).

Confirmatory factory analysis (CFA) was performed using Lavaan package version 06.6 (Rosseel 2012). The CFA models were estimated using the robust-weighted least square (WLMSV) estimator due to the highly skewed categorical data (Flora and Curran 2004). Missing data were dealt with using pair-wise deletion. Model fit was assessed using robust versions of Chi-square, the comparative fit index (CFIrobust), the root mean square error of approximation (RMSEArobust) and SRMRrobust. CFI values greater than 0.90 together with RMSEA values of less than 0.08 and SRMR of less than 0.08 were considered acceptable (Brown 2006), whereas CFI values above 0.95 and RMSEA of below 0.06 and SRMR of less than 0.05 were preferred (Hu and Bentler 1999). Measurement invariance across gender, age (over 50 versus under 50 years of age) and household income (over versus below 500,000 NOK) (1 € = NOK 10.67, as of 4 July, 2020), respectively, was tested by comparing a model where the fit of a model of which the factor loadings and thresholds were equal across groups (strong invariance) with the fit of a model of which the same parameters (except for identification items) were free to vary (configural invariance). If the change in CFI was > 0.002 (Meade et al. 2008), the factor loadings and thresholds were relaxed in tandem for one and one item at the time (see Muthén and Muthén 2017 p.546). Items were flagged as functioning differently across groups if delta chi-squared was significant. Finally, the covariation between factors was constrained to be equal across groups to explore whether this led to a significantly poorer fit as measured by delta chi-squared. The significant level was set at *p* < 0.01 in these invariance tests to adjust for family-wise error rate as a means to avoid potential type I errors when performing multiple tests.

## Results

In total, 1063 (73%) were available for analysis; 588 (55%) were female and 475 (45%) male. The age distribution was quite even with the highest percentage in the group aged 50–59 years (21%), and the lowest among those aged 18–29 years (12%). The majority had education at university level (60%), and nearly half of them were full-time workers (50%) (Table 1). Five questions described coronavirus-related actions over the last 4 weeks. Half of the respondents had been working from home or home schooling (50%). Most answered that they had tried to keep a distance from people around them (88%), while 5.8% had been ill with suspected, possible or confirmed COVID-19 infection or been in a household with a person with suspected, possible or confirmed COVID-19 infection (5.1%) (Table 2).

**Table 1:** Socio-demographic data (*N* = 1063)

Variables	Frequency	Percentage
Gender		
Female	588	55.3
Male	475	44.7
Age categories (in years)		
18–29	129	12.1
30–49	150	14.1
40–49	186	17.5
50–59	227	21.4
60–69	201	18.9
70–79	170	16.0
Education (*n* = 989)		
Primary and lower secondary school (10 years)	65	6.1
Upper secondary school	291	27.4
University less than 4 years	230	21.6
University ≥ 4 years	403	37.9
Household income (NOK^a^) (including benefits) (*n* = 899)		
< 250,000	117	13.0
250,000–500,000	384	42.7
> 500,000	398	44.3
Occupational status before COVID-19 outbreak*		
Full-time work	562	47.1
Disability benefits	58	5.5
Home worker	11	1
On sick leave	15	1.4
Part-time work	103	9.7
Retired	227	21.4
Social benefits	5	0.5
Temporarily laid off	4	0.4
Unemployed	17	1.6
Student/ military service	69	6.5
None of the above	6	0.6
Change in working conditions after COVID-19 outbreak*		
New job or other job position	28	2.6
Receive wages, but cannot do my work	23	2.2
Temporarily laid off	75	7.1
Unemployed	7	0.7
Working from home	379	35.7
None of the above	203	19.1
Sector of employment		
Healthcare worker	158	14.9
Industry/petroleum	73	6.9
Retail	44	4.1
Teaching/university	112	10.5
Transport	31	2.9
None of the above	76	7.1
Other	212	20
Norwegian citizen		
Yes	1007	95.73
No	56	5.27

^a^NOK = Norwegian kroner (1 € = NOK 10.67) (as of 4 July, 2020)

*More than one alternative possible

**Table 2:** Coronavirus-related actions over the last 4 weeks (*N* = 1048)

Actions	Frequency	Percentage
Have been in quarantine	187	17.8
Have been ill with suspected, possible or confirmed COVID-19 infection	62	5.9
Have been working from home or home schooling	522	49.8
Have been in household with a person with suspected, possible or confirmed COVID-19 infection	53	5.1
Have tried to keep a distance from people around me	919	87.7

### Fear of COVID-19

As shown in Table 3, less than 10% of the respondents either agreed or strongly agreed with most of the FCV-19S items suggesting that the level of fear of COVID-19 is generally low in this population. For three of the items, all of which assessed somatic symptoms (q3, q6, and q7), only two or less strongly agreed with the item. Two items were somewhat less asymmetric; however, since 15% and 33% either agreed or strongly agreed that “they were most afraid of corona” (q1) and that “it made them uncomfortable to think about corona” (q2), respectively. The asymmetric response is also highlighted in Figure S1, which shows that 14.5% strongly disagreed on all the items and therefore had the lowest possible sum score on the FCV-19S. Apart from these extremely low scorers, the distribution of the total scale score was rather close to a normal distribution (skewness = 0.83, kurtosis = 0.55).

**Table 3:** Frequency distribution of answers on Fear of COVID-19 Scale in a Norwegian population (*N* = 1063)

	Strongly disagree	Disagree	Neutral	Agree	Strongly agree
Questions	*n* (%)	*n* (%)	*n* (%)	*n* (%)	*n* (%)
q1 I am most afraid of corona	256 (24.1%)	372 (35.1%)	278 (26.2%)	134 (12.6%)	21 (2%)
q2 It makes me uncomfortable to think about corona	217 (20.5%)	239 (22.6%)	254 (24.0%)	319 (30.1%)	30 (2.8%)
q3 My hands become clammy when I think about corona	724 (68.4%)	252 (23.8%)	64 (6.0%)	17 (1.6%)	2 (0.2%)
q4 I am afraid of losing my life because of corona	527 (49.8%)	311 (29.4%)	134 (12.7%)	70 (6.6%)	17 (1.6%)
q5 When watching news and stories about corona on social media, I become nervous or anxious	430 (40.6%)	343 (32.4%)	180 (17.0%)	97 (9.2%)	10 (0.9%)
q6 I cannot sleep because I’m worrying about getting corona	769 (72.9%)	216 (20.5%)	59 (5.6%)	11 (1.0%)	0 (0%)
q7 My heart races or palpitates when I think about getting corona	766 (72.3%)	231 (21.8%)	47 (4.4%)	14 (1.3%)	1 (0.1%)

### Reliability

Both Cronbach’s alpha (0.88) and Omega alphaTotal (*ωt* = 0.91) indicate that the scale has a very good internal consistency. However, the Omega alphaHiercical (ωh) coefficient suggests that the general factor is not very strong explaining only 69% of the total variance.

### Structural Validity—Dimensionality Assessment

**Fig. 2:**
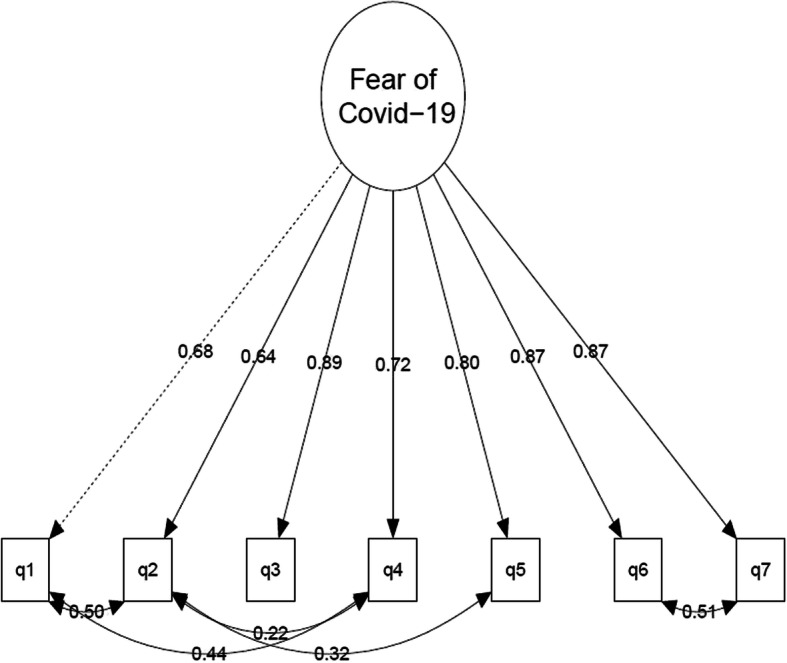
Modified one-factor model (M1b). q1–q7 refer to questions in FSV-19S as written in full Table 3

The one-factor model (M1) has an unsatisfactory fit to the data (Table 4). While the CFI was good (> 0.95), both SMRS and particularly RMSEA were above conventional cut-offs for acceptable fit (> 0.08). Using modification indices, we allowed for correlations between 5 pairs of residuals (local dependencies) to obtain an acceptable fit (M1b). Figure 2 shows that these local dependencies are far from negligible (*r* = 0.22–0.51). The inclusion of these local dependencies had a clear impact on the factor loadings of two items as the factor loading of q1 decreased from 0.81 to 0.68 from M1 to M1b while the factor loading of q2 decreased from 0.78 to 0.64.

**Table 4:** Fit of models

	Chi-squared robust	CFI robust	RMSEA robust	SRMS robust
M1: One-factor model	359.05 df = 14, *p* < 0.001	0.972	0.152 (0.139, 0.166)	0.082
M1b with five correlated pairs of residuals	41.59, df = 9, *p* < 0.001	0.997	0.058 (0.041, 0.077)	0.021
M2: Two-factor model	197.24, df = 13, *p* < 0.001	0.985	0.116 (0.102, 0.130)	0.051
M2b: Alternative two-factor model with four correlated pairs of residuals	32.75, df = 9, *p* < 0.001	0.998	0.050 (0.032, 0.069)	0.019
M3: Second-order model	32.75, df = 9, *p* < 0.001	0.998	0.050 (0.032, 0.069)	0.019

**Fig. 3:**
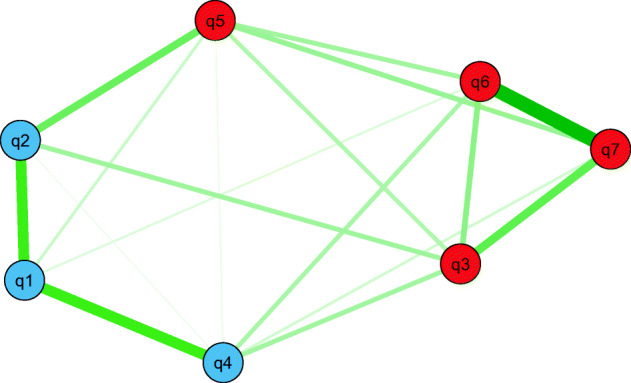
Explorative dimensionality assessment by Explorative Graph Analysis detecting the number of factors of the Fear of COVID-19 Scale. The colour of the nodes represents dimensions while the thickness of the lines represents the size of the partial correlation. q1–q7 refer to questions in FSV-19S as written in full Table 3

**Fig. 4:**
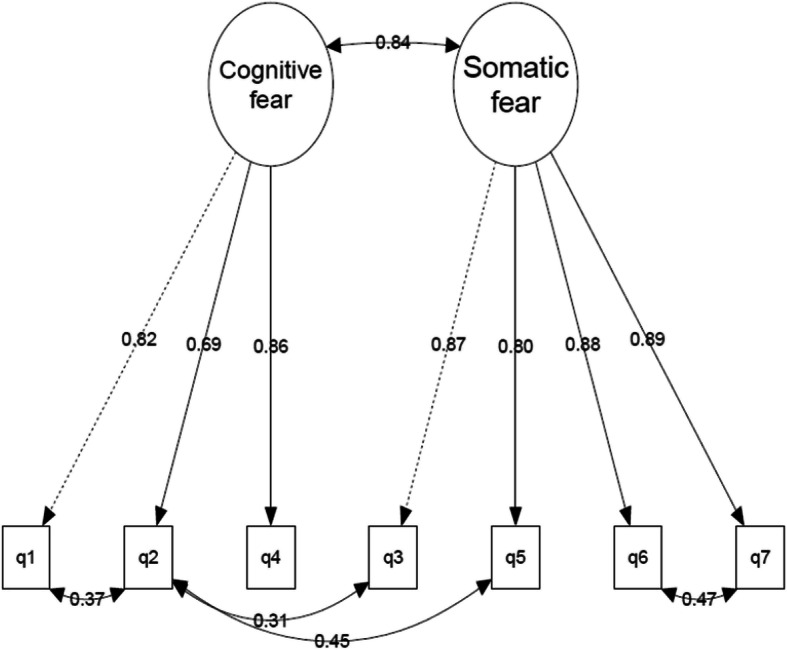
Correlated two-factor model of cognitive and somatic fear with correlated residuals (M2b). q1–q7 refer to questions in FSV-19S as written in full Table 3

Due to the poor fit of the unmodified unidimensional model, we conducted an exploratory graph analysis (EGA) to detect the number of factors in the scale. As shown in Fig. 3, the EGA analysis suggests that the COVID-19 scale consists of two dimensions. Given their content, we interpret these dimensions as reflecting cognitive fear (q1, q2, and q4) and somatic fear (q3, q5, q6, and q7). The robustness of the factor structure was supported by EGA bootstrap analyses (1000 draws with replacements), using both “glasso” and “TMFG” estimators (Christensen and Golino 2019). Although a CFA model based on these two factors (M2) had a significantly better fit than the unmodified unidimensional model (Δ*Χ*^2^ = 98.71, df = 1, *p* < 0.001), the RMSEA index was unsatisfactory also in this model (0.116). To obtain an acceptable fit for this index (M2b), we had to allow for four rather large local dependencies (q1 & q2, q2 & q5, q2 & 3, and q6 & q7) (Fig. 4).

The correlated two-factor model was measurement invariant across gender and household income but not age. Q4 (I am afraid of losing my life because of corona) functioned somewhat differently for individuals above 50 years than younger people (Δ*X*^2^ = 13.01, df = 4, *p* < 0.01) as the above 50-year-old group seemed to be somewhat more likely to have a higher score on this item than the younger age group with similar level of cognitive fear. Allowing the loading and thresholds of this item to be free to vary across these age groups had only a minor impact (− 0.04 standard deviation units) on the differences in latent means on the cognitive fear factor (0.58 versus 0.62 standard deviation units higher cognitive fear in the older age group). The covariation between the cognitive and somatic fear factors was found to be invariant across gender, age, and household income.

**Fig. 5:**
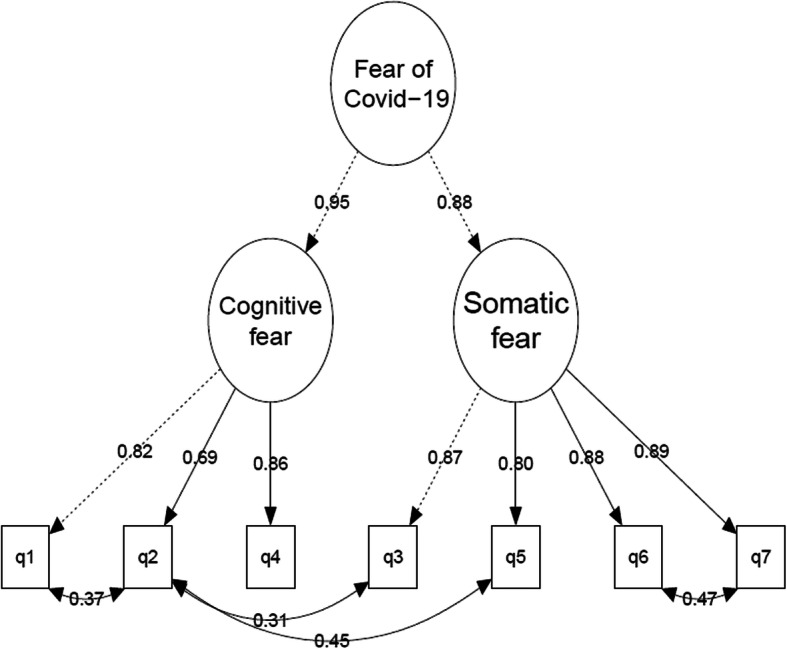
Second-order mode of cognitive and somatic fear as first-order factors and general fear of COVID-19 as a secondary factor (M3). q1–q7 refer to questions in FSV-19S as written in full Table 3

It should be noted that the two factors were highly correlated (*r* = 0.84). A mathematically identical model (with the same model fit) but with a conceptually different meaning is shown in Fig. 5. In this second-order hierarchical model, the large correlation between the factors is transformed into a second-order general fear of COVID-19 factor of which the two latent factors serve as indicators (loadings of the general factor constrained to be equal to identify the model). This model suggests that, even if there are two separate first-order COVID-19 fear factors (cognitive and somatic fear), both are strongly determined by the second-order of general fear of COVID-19 factor.

### Concurrent Validity and Association with Other Variables

The sum score of FCV-19S correlated significantly with other constructs like poorer mental health (SCL-10) (*r* = 0.35, *p <* 0.001) and lower life satisfaction (*r* = − 0.19, *p* < 0.001) (Table 5). A higher FCV-19S score was positively associated with being female, older age groups, and lower socioeconomic status (lower education and income). FCV-19S was statistically significantly associated with questions like being worried that they themselves and their family could be infected by the virus, as well as worries linked to the possibility of experiencing financial problems or losing or being laid off from their job. Similarly, FCV-19S correlated with one question concerning coronavirus-related actions over the last 4 weeks before the survey, e.g., a negative correlation with working from home or home schooling. The correlation coefficients were in most cases smaller than ± 0.20, except for the FCV-19S correlation with mental health problems (SCL10, *r* = 0.35) and worries about themselves and their family possibly getting infected (*r* = 0.34–0.64).

**Table 5:** Correlation coefficients for Fear of COVID-19 Scale (FCV-19S) and the subscales with socio-demographics, the SCL-10 scale, coronavirus-related worry questions, and coronavirus-related actions over the last 4 weeks

Variables	Fear of COVID-19 Total	Fear of COVID-19 Cognitive	Fear of COVID-19 Somatic
Socio-demographics Male (*n* = 1048)	−0.14***	−0.12***	−0.14***
Age (*n* = 1048)	0.20***	0.23***	0.12***
Household income (*n* = 887)	−0.09**	−0.03	−0.15***
Education (*n* = 976)	−0.16***	−0.13***	−0.16***
SCL-10 (*n* = 959)	0.35***	0.26***	0.38***
Satisfaction with life (*n* = 1048)	−0.19***	−0.14***	−0.21***
General health (*n* = 1047)	−0.23***	−0.20***	−0.22***
Meaningfulness (*n* = 1047)	−0.18***	−0.14***	−0.19***
Coronavirus-related worries, Worried that			
Some of my next of kin could get infected (*n* = 1044)	0.45***	0.44***	0.38***
I could get infected (*n* = 1043)	0.64***	0.65***	0.50***
Some of the elderly in the family could get infected (*n* = 1040)	0.34***	0.34***	0.29***
My child/children fall ill with coronavirus (*n* = 791)	0.52***	0.51***	0.43***
The outbreak could cause me to be laid off or lose my job (*n* = 1013)	0.09**	0.05	0.11***
The outbreak could lead to poorer finances (*n* = 1042)	0.13***	0.10***	0.15***
I do not let the fuss around the coronavirus get to me (n = 1048)	−0.34***	−0.33***	−0.28***
Coronavirus-related actions over the last 4 weeks			
Quarantine (*n* = 1048)	−0.01	−0.01	0.00
Have been ill with suspected, possible or confirmed COVID-19 infection (*n* = 1048)	−0.03	−0.03	−0.02
Have worked from home or home schooling (*n* = 1048)	−0.14***	−0.13***	−0.12***
Have been in a household with a person with suspected, possible or confirmed COVID-19 infection (*n* = 1048)	−0.05	−0.07*	−0.03
Have tried to keep a distance from people around me (*n* = 1048)	−0.02	0.01	−0.05

*SCL* Symptom Check List

*p < 0.05

**p < 0.01

***p < 0.001

The cognitive (*r* = 0.91) and somatic (*r* = 0.90) subscales had similar associations with the sum score of FCV-19S, as well as other constructs. This was also the case for most socio-demographic variables (Table 5). Only “Household income” and “The outbreak could cause me to be laid off or lose my job” were not statistically significantly associated with the cognitive subscale. This subscale also tended to have a somewhat weaker correlation with mental health problems (SCL-10, *r* = 0.26 vs. 0.38) and life satisfaction (*r* = − 0.14 vs. − 0.21) than the somatic subscale. The somatic subscale had a somewhat weaker correlation with age (*r* = 0.12 vs. 0.20) and worries about themselves and their family possibly getting infected (*r* = 0.29–0.50 vs. 0.34–0.65) than the cognitive subscale.

## Discussion

Exploratory graph analysis and confirmatory factor analysis found support for a two-factor model (cognitive and somatic fear), which were highly correlated (*r* = 0.84). Therefore, it might be most fruitful to present the FCV-19S by using a hierarchical model where a general factor is a second-order factor accounting for the high correlation between the first-order factors (cognitive and somatic fear). This model is theoretically meaningful and supports use of the FCV-19S sum score.

The internal consistency reliability for the Norwegian version of the FCV-19S was found to be very good when assessed by Cronbach’s alpha (0.88). This is in line with studies from other countries (Ahorsu et al. 2020; Alyami et al. 2020; Bitan et al. 2020; Reznik et al. 2020; Sakib et al. 2020; Satici et al. 2020; Soraci et al. 2020; Tsipropoulou et al. 2020; Winter et al. 2020). Although Omega alphatotal is often regarded as a better alternative than Cronbach’s alpha (McNeish 2018; Revelle and Condon 2018), it produced very similar results in the present study (0.91). It should be noted that although the items are ordinal, they were treated as continuous variables in the reliability analyses, as this is probably most appropriate when assessing the reliability of simple unweighted sum scores (Gustafsson and Aberg-Bengtsson 2010; Revelle and Condon 2018). A potential limitation of both these reliability coefficients is that they assume that the scale is unidimensional. As mentioned in the introduction section, the results from some studies have indicated that the FCV-19S might not be strictly unidimensional (Alyami et al. 2020; Soraci et al. 2020). Accordingly, the Omega hierarchical coefficient (*ωh*) was found to be only 0.69, which is at the boundary of what is conventionally regarded as acceptable reliability (Nunnally and Bernstein 1994). The Omega hierarchical coefficient is model-based and assesses the reliability of the general factor in either a bi-factor or a hierarchical second-order factor structure (Savalei and Reise 2019). Our results thus suggest that when assessing the reliability of the latent variable representing the common variance across all the items in FCV-19S, the often-used Cronbach’s alpha (and *ωt*) will lead to a somewhat inflated result. Thus, researchers are advised to include ωh in future reliability assessments of this scale.

Despite some previous studies have shown unidimensional structure for FCV-19S, there are some inconsistencies in their underlying factor structures in other versions, including a bi-dimensional structure representing physiological and emotional responses (Bitan et al. 2020; Reznik et al. 2020; Pakpour et al. 2020b; Pakpour et al. 2020c). Furthermore, RMSEA values above the preferred cut-off value of 0.06 were identified (Alyami et al. 2020; Tsipropoulou et al. 2020; Winter et al. 2020). In line with this, the unidimensional model showed poor fit in our study and several correlated residuals had to be included to obtain acceptable fit.

The results from the EGA analysis suggest that the FCV-19S consists of two dimensions: cognitive fear and somatic fear. These dimensions are similar to those detected in the Eastern European and Israeli version of the scale (Bitan et al. 2020; Reznik et al. 2020; Pakpour et al. 2020b) except for item 5 (When watching news and stories about corona on social media, I become nervous or anxious), which was categorised as a somatic factor in our study. One reason for this difference could be that the Eastern European and Israeli study used PCA to assess dimensionality and assumed that the factors were orthogonal to each other.

After allowing for three correlated pairs of residuals, the two-factor model had a good fit to the data. Our analyses indicate, moreover, that multidimensionality has an impact on the size of the factor loadings (M1 versus M1b). This means that in a traditional IRT/RASCH model, when assuming unidimensionality, the parameter values will probably be wrong. This bias will subsequently be problematic if factor loadings or discriminative parameters (IRT) are used as baseline for example in computer adaptive testing, calculation of reliability, or when making any changes to the scale by removal of items based on the size of these parameters.

The correlated two-factor model was found to be measurement invariant across gender and household income and age with one exception. This study suggests that the item (q4), which measures whether one is afraid of losing one’s life due to corona, functions somewhat differently depending on whether one is above 50 years or not. The consequence of this is that individuals above 50 years old seem to be more likely to endorse this item than younger people with a similar degree of cognitive fear of COVID-19. It is perhaps not surprising that this item functions somewhat differently dependent on age given the fact that the risk of death due to COVID-19 is much lower for people younger than 50. Our analyses suggest that this lack of invariance has negligible effect on mean age group differences on the cognitive fear factor which supports the inclusion of this item when analysing this subscale.

It is worth noting that the correlation between the cognitive and somatic fear factors was very large (*r* = 0.84) in our study, so large in fact as to question their discriminant validity as two separate factors (Brown 2006). We regard this large correlation as support for a second-order hierarchical model in which two latent factors (somatic and cognitive fear) act as indicators of a second-order general fear of COVID-19 factor. Distinguishing between cognitive and somatic components of anxiety has been supported (Schoen and Holtzer 2017), and the general factor supports use of the FCV-19S sum score. Given the large correlation between the cognitive and somatic factors, we believe that it is often sufficient to treat the scale as essential unidimensional and use the sum score. In some cases however, differencing between cognitive and somatic fear might provide unique information that might turn out to be of importance for further understanding of fear of COVID-19.

In previous validation studies, the sum score of FCV-19S has shown good concurrent validity with other constructs measuring for example anxiety and depression (Ahorsu et al. 2020; Alyami et al. 2020; Bitan et al. 2020; Reznik et al. 2020; Sakib et al. 2020; Satici et al. 2020; Soraci et al. 2020; Tsipropoulou et al. 2020; Winter et al. 2020). Our results were in line with these studies and confirmed sound concurrent validity as the FCV-19S sum score was positively correlated with SCL-10 and negatively correlated with satisfaction with life. FCV-19S was statistically significantly associated with one of the coronavirus-related actions over the last 4 weeks before the survey, namely that fear of COVID-19 had a negative correlation with working from home or home schooling. It is not surprising that those working from home report less fear of COVID-19 than those who do not have this opportunity, as they probably feel that they have a lower risk of catching the virus. Fear of COVID-19 has been associated with people practising social distancing by maintaining the two-metre rule (i.e. maintaining a 2-m distance from other people when in public places), and only engaging in physical activity in outdoor places that were readily accessible on foot (Winter et al. 2020). Interestingly, Harper et al. (2020) also reported a positive correlation between FCV-19S scores and participants’ judgement of the degree to which several behaviours and practices had changed due to the pandemic (e.g. hand washing and care of children and the elderly). Furthermore, the relationship between fear and preventive behaviours has been explored (Chang et al. 2020b). In addition, fear of COVID-19, as measured by the FCV-19S, is found to be a mediator in the association between problematic social media use and distress and insomnia (Chang et al. 2020b; Lin et al. 2020), thus given further support for its construct validity.

The cognitive and somatic subscales had in most cases significant and similar degree of associations with the other variables as the total scale. However, in some cases, the use of these subscales provided possible insight in rather unique relations. “Household income” and “The outbreak could cause me to be laid off or lose my job” were only statistically significantly associated with the somatic subscale. This subscale also had a somewhat stronger association with mental health problems than the cognitive subscale. The cognitive fear subscale, on the other hand, tended to have a somewhat stronger correlation with age as well as worries about family and themselves getting infected by the virus. According to Cohen’s criteria (1988), most of the effect sizes in this study can be classified as small. However, regarding the FCV-19S relationship with mental health problems (SCL10) and worries about themselves and their family possibly getting infected, the effect sizes were medium to large.

As many as 14.5% strongly disagreed with all the FCV-19S items and therefore had the lowest possible sum score. This is very close to the conventional cut-off for a floor effect (Terwee et al. 2007) (Figure S1). However, the results of the study should be interpreted in light of the prevalence of COVID-19 in the country at the time of the data collection. In Norway, the first cases of COVID-19 were confirmed in February 2020. The peak of the outbreak was during March and April. As of 1 July, the total number of confirmed cases was 8912 and there had been 251 deaths. In the city of Bergen, the second largest city in Norway, 575 cases and 31 deaths have been confirmed so far (Norwegian Institute of Public Health 2020). In the data collection period, 4–23 June, only five citizens tested positive for COVID-19. Apart from some extremely low scorers, which is not unexpected given the data collection period, the distribution of the total scale score seems to be rather close to a normal distribution.

## Strengths and Limitations

The sampling technique is a strength of this study. Individuals were drawn from the Contact and Reservation Registry through the Norwegian Digitalisation Agency for inclusion in a larger COVID-19 study, and then randomly selected to participate in a follow-up survey in which we included the FCV-19S. The response rate was 73%. To date, most other validation studies of the FCV-19S are convenience studies, using snowball sampling or social media networks. In addition, our study had a broad age and gender distribution. However, as with all large-scale validation studies, this study also has inherent shortcomings. The study design was cross-sectional, and therefore, this study did not examine the stability of the FCV-19S over time. The measurement time may not have been ideal since COVID-19 was decreasing at the time, and fear was therefore expected to do the same. However, this is uncertain as the easing of restrictions may increase fear in some people. The data showed close to normal distribution.

## Conclusion

The Norwegian version of the FCV-19S showed not to be strictly unidimensional. We found support for a bi-dimensional fit with two highly correlated factors representing cognitive and somatic fear. Due to the high correlation, we believe it is fruitful to regard the two latent factors as indicators of a second order general factor. This general factor seems to have a very strong impact on cognitive and somatic fear which support use of the FCV-19S sum score. We found that the total scale correlated meaningfully with other variables. Further research is needed to investigate whether the subscales provide important information over and above what can be determined from the sum score of FCV-19S.

## Supplementary Information

Figure S1. Distribution of FCV-19S sum scores (JPG 59 kb)

## References

[CR1] Ahorsu, D. K., Lin, C. Y., Imani, V., Saffari, M., Griffiths, M. D., & Pakpour, A. H. (2020). The fear of COVID-19 scale: development and initial validation. *International Journal of Mental Health and Addiction*. 10.1007/s11469-020-00270-8. 10.1007/s11469-020-00270-8 PMC7100496 32226353

[CR2] Alyami M, Henning M, Krägeloh CU, Alyami H (2020). Psychometric evaluation of the Arabic version of the fear of COVID-19 scale. International Journal of Mental Health and Addiction.

[CR3] Beaton DE, Bombardier C, Guillemin F, Ferraz MB (2000). Guidelines for the process of cross-cultural adaptation of self-report measures. Spine.

[CR4] Bitan DT, Grossman-Giron A, Bloch Y, Mayer Y, Shiffman N, Mendlovic S (2020). Fear of COVID-19 scale: psychometric characteristics, reliability and validity in the Israeli population. Psychiatry Research.

[CR5] Brown TA (2006). *Confirmatory factor analysis for applied res*earch.

[CR6] Chang, K.-C., Hou, W.-L., Pakpour, A. H., Lin, C.-Y., & Griffiths, M. D. (2020a). Psychometric testing of three COVID-19-related scales among people with mental illness. *International Journal of Mental Health and Addiction*. 10.1007/s11469-020-00361-6. 10.1007/s11469-020-00361-6 PMC7354353 32837442

[CR7] Chang K-C, Strong C, Pakpour AH, Griffiths MD, Lin C-Y (2020). Factors related to preventive COVID-19 infection behaviors among people with mental illness. Journal of the Formosan Medical Association.

[CR8] Chen I-H, Chen C-Y, Pakpour AH, Griffiths MD, Lin C-Y (2020). Internet-related behaviors and psychological distress among schoolchildren during COVID-19 school suspension. Journal of the American Academy of Child and Adolescent Psychiatry.

[CR9] Christensen, A. P., & Golino, H. (2019). *Estimating the stability of the number of factors via bootstrap Exploratory Graph Analysis: a tutorial*. PsyArXiv. 10.31234/osf.io/9deay.

[CR10] Cohen, J. (1988). *Statistical power analysis for the behavioral sciences*. 2nd Edition. New York.

[CR11] Flora DB, Curran PJ (2004). An empirical evaluation of alternative methods of estimation for confirmatory factor analysis with ordinal data. Psychological Methods.

[CR12] Golino, H., & Christensen, A. P. (2019). EGAnet: Exploratory Graph Analysis: a framework for estimating the number of dimensions in multivariate data using network psychometrics. URL: https://CRAN.R-project.org/package= EGAnet. R package version 0.9.3. Accessed 1 July 2020.

[CR13] Golino H, Shi D, Christensen AP, Garrido LE, Nieto MD, Sadana R, Thiyagarajan JA, Martinez-Molina A (2020). Investigating the performance of Exploratory Graph Analysis and traditional techniques to identify the number of latent factors: a simulation and tutorial. Psychological Methods.

[CR14] Gustafsson JE, Aberg-Bengtsson L, Embretson SE (2010). Unidimensionality and the interpretability of psychological instruments. Measuring psychological constructs.

[CR15] Harper, C. A., Satchell, L. P., Fido, D., & Latzman, R. D. (2020). Functional fear predicts public health compliance in the COVID-19 pandemic. *International Journal of Mental Health and Addiction*. Epub ahead of print. 10.1007/s11469-020-00281-5. 10.1007/s11469-020-00281-5 PMC7185265 32346359

[CR16] Hesbacher PT, Rickels K, Morris RJ, Newman H, Rosenfeld H (1980). Psychiatric illness in family practice. The Journal of Clinical Psychiatry.

[CR17] Hossain MM, Tasnim S, Sultana A, Faizah F, Mazumder H, Zou L, McKyer LE, Ahmed HU, Ma P (2020). Epidemiology of mental health problems in COVID-19: a review. F1000Research.

[CR18] Hu LT, Bentler PM (1999). Cutoff criteria for fit indexes in covariance structure analysis: Conventional criteria versus new alternatives. Structural Equation Modeling.

[CR19] Lin C-Y, Broström A, Griffiths MD, Pakpour AH (2020). Investigating mediated effects of fear of COVID-19 and COVID-19 misunderstanding in the association between problematic social media use and distress/insomnia. Internet Interventions.

[CR20] Lipsitch M, Swerdlow DL, Finelli L (2020). Defining the epidemiology of Covid-19 – Studies needed. New England Journal of Medicine.

[CR21] McNeish D (2018). Thanks coefficient alpha, we’ll take it from here. Psychological Methods.

[CR22] Meade AW, Johnson EC, Braddy PW (2008). Power and sensitivity of alternative fit indices in tests of measurement invariance. Journal of Applied Psychology.

[CR23] Muthén LK, Muthén BO (2017). Mplus user’s guide.

[CR24] Norwegian Institute of Public Health. (2020). *Coronavirus disease—advice and information*. Retrieved from https://www.fhi.no/en/id/infectious-diseases/coronavirus/. Accessed 1 July 2020.

[CR25] Nunnally, J. C., & Bernstein, I. H. (1994). *Psychometric theory* (3rd ed.). McGraw-Hill. Accessed 1 July 2020.

[CR26] Pakpour AH, Griffiths MD, Chang K-C, Chen Y-P, Kuo Y-J, Lin C-Y (2020). Assessing the fear of COVID-19 among different populations: a response to Ransing et al. Brain, Behavior, and Immunity.

[CR27] Pakpour AH, Griffiths MD, Lin C-Y (2020). Assessing the psychological response to the COVID-19: a response to Bitan et al. “fear of COVID-19 scale: Psychometric characteristics, reliability and validity in the Israeli population”. Psychiatry Research.

[CR28] Pakpour, A. H., Griffiths, M. D., & Lin, C.-Y. (2020c). Assessing psychological response to the COVID-19: the fear of COVID-19 scale and the COVID stress scales. *International Journal of Mental Health and Addiction*. 10.1007/s11469-020-00334-9. 10.1007/s11469-020-00334-9 PMC7259433 32837424

[CR29] R Development Core Team. (2020). *R: a language and environment for statistical computing*.

[CR30] R Foundation for Statistical Computing (n.d.). Vienna, Austria: R Foundation for Statistical, Computing. R Foundation for Statistical Computing, Vienna, Austria. ISBN 3–900051–07-0, URL http://www.R-project.org. Accessed 1 July 2020.

[CR31] Ransing, R., Ramalho, R., Orsolini, L., Adiukwu, F., Gonzalez-Diaz, J. M., Larnaout, A., Pinto da Costa, M., Grandinetti, P., Bytyçi, D. G., Shalbafan, M., Patil, I., Nofal, M., Pereira-Sanchez, V., & Kilic, O. (2020). Can COVID-19 related mental health issues be measured? *Brain, Behavior, and Immunity*. May 26:S0889–1591(20)30932–6. 10.1016/j.bbi.2020.05.049. 10.1016/j.bbi.2020.05.049 PMC7248629 32470593

[CR32] Revelle, W. (2014). *Package ‘psych’*. Retrieved from http://cran.r-project.org/web/packages/psych/psych.pdf. Accessed 1 July 2020.

[CR33] Revelle W, Condon DM, Irwing P, Booth T, Hughes DJ (2018). Reliability. *The Wiley handbook of psychometric testing: a multidisciplinary reference on survey, scale and test development*.

[CR34] Reznik, A., Gritsenko, V., Konstantinov, V., Khamenka, N., & Isralowitz, R. (2020). COVID-19 fear in Eastern Europe: validation of the fear of COVID-19 scale. *International Journal of Mental Health and Addiction*, Epub ahead of print. 10.1007/s11469-020-00283-3. 10.1007/s11469-020-00283-3 PMC7217343 32406404

[CR35] Rosseel Y (2012). lavaan*:* an R package for structural equation modeling. Journal of Statistical Software.

[CR36] Sakib, N., Bhuiyan, A. K. M. I., Hossain, S., Mamun, A. F., Hosen, I., et al. (2020). Psychometric validation of the Bangla fear of COVID-19 scale: confirmatory factor analysis and Rasch analysis. *International Journal of Mental Health and Addiction*, Epub ahead of print. 10.1007/s11469-020-00289-x. 10.1007/s11469-020-00289-x PMC7213549 32395096

[CR37] Satici, B., Gocet-Tekin, E., Deniz, M. E., & Satici, S. A. (2020). Adaptation of the fear of COVID-19 scale: its association with psychological distress and life satisfaction in Turkey. *International Journal of Mental Health and Addiction*, Epub ahead of print. 10.1007/s11469-020-00294-0. 10.1007/s11469-020-00294-0 PMC7207987 32395095

[CR38] Savalei, V., & Reise, S. P. (2019). Don’t forget the model in your model-based reliability coefficients: a reply to McNeish (2018). *Collabra: Psychology, 5*(1), 36. 10.1525/collabra.247

[CR39] Schoen BC, Holtzer R (2017). Differential relationships of somatic and cognitive anxiety with measures of processing speed in older adults. Neuropsychology, development and cognition. Section B, Aging, neuropsychology and cognition.

[CR40] Soraci, P., Ferrari, A., Abbiati, F. A., Del Fante, E., De Pace, R., Urso, A., & Griffiths, M. D. (2020). Validation and psychometric evaluation of the Italian version of the fear of COVID-19 scale. *International Journal of Mental Health and Addiction*. 10.1007/s11469-020-00277-1. 10.1007/s11469-020-00277-1 PMC7198091 32372892

[CR41] Strand BH, Dalgard OS, Tambs K, Rognrerud M (2003). Measuring the mental health status of the Norwegian population: a comparison of/he instruments SCL-25, SCL-10, SCL-5 and MHl-5. Nordic Journal of Psychiatry.

[CR42] Terwee CB, Bot SD, de Boer MR, van der Windt DA, Knol DL (2007). Quality criteria were proposed for measurement properties of health status questionnaires. Journal of Clinical Epidemiology.

[CR43] Tsipropoulou, V., Nikopoulou, V. A., Holeva, V., Nasika, Z., Diakogiannis, I., Sakka, S., Kostikidou, S., Varvara, C., Spyridopoulou, E., & Parlapani, E. (2020). Psychometric properties of the Greek version of FCV-19S. *International Journal of Mental Health and Addiction*. 10.1007/s11469-020-00319-8. 10.1007/s11469-020-00319-8 PMC7250285 32837420

[CR44] Winter, T., Riordan, B. C., Pakpour, A. H., Griffiths, M. D., Mason, A., Poulgrain, J. W., & Scarf, D. (2020). Evaluation of the English version of the fear of COVID-19 scale and its relationship with behavior change and political beliefs. *International Journal of Mental Health and Addiction*. 10.1007/s11469-020-00342-9. 10.1007/s11469-020-00342-9 PMC7295324 32837431

[CR45] Zortea, T. C., Brenna, C. T. A., Joyce, M., McClelland, H., Tippet, M., Tran, et al. (2020). The impact of infectious disease-related public health emergencies on suicide, Suicidal Behavior, and Suicidal Thoughts. *Crisis*, 1–14. 10.1027/0227-5910/a000753. 10.1027/0227-5910/a000753 PMC8689932 33063542

