# Supplementary figures and images for: Psychometric Properties of the Norwegian Version of the Fear of COVID-19 Scale
Source: Int J Ment Health Addict. 2021 Jan 20;20(3):1446–64. doi: 10.1007/s11469-020-00454-2 (PMC7816751; doi:10.1007/s11469-020-00454-2)

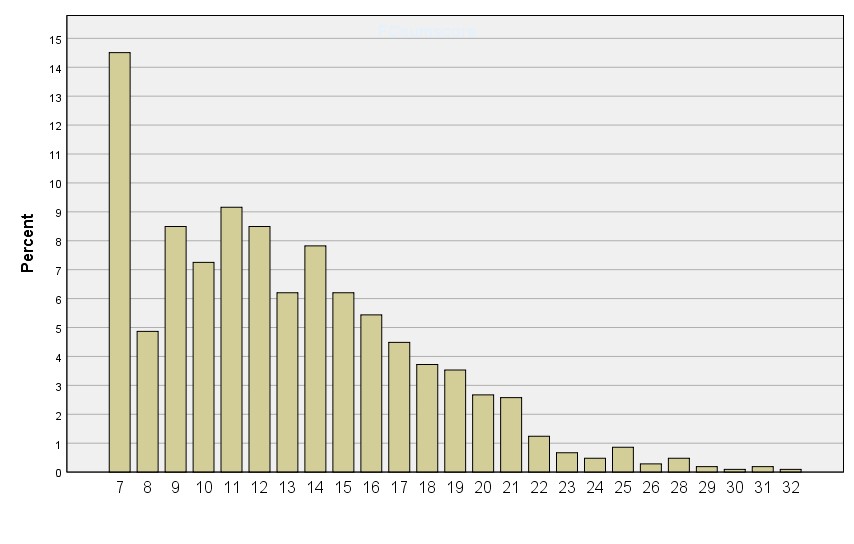

Supplement: Supplementary file 1 — Distribution of FCV-19S sum scores (JPG 59 kb) [file 11469_2020_454_MOESM1_ESM.jpg]
